# Sex Differences in Physical Activity of US children at age 13 months: Child and Mother Physical Activity Study (CAMPAS)

**DOI:** 10.21203/rs.3.rs-4552035/v1

**Published:** 2024-06-26

**Authors:** Soyang Kwon, Sarah Welch, Selin Capan

**Affiliations:** Ann and Robert H Lurie Children’s Hospital; Northwestern University; Northwestern University

## Abstract

**Background:**

Lower physical activity (PA) has been observed in females compared to males among preschool-aged and older children. However, the timing of when the sex gap emerges is unclear. The purpose of this study was to investigate whether females have lower PA levels than males in the early toddler age and to explore whether gross motor competency and PA parenting practices might explain a sex difference in PA.

**Methods:**

The study design was cross-sectional. Participants were a community-based sample of 137 children aged 10–16 months residing in US Midwest urban/suburban area. Participants’ mothers completed a survey that contained a demographic questionnaire, the Ages and Stages Questionnaire gross motor competency subscale, and a PA parenting practices questionnaire. Participating children wore an ActiGraph accelerometer on their hip for 7 days. Accelerometer-measured time spent in moderate- and vigorous intensity PA (MVPA; minutes/day) and in total PA (minutes/day) were calculated. Multivariable linear regression analysis was conducted to predict MVPA and total PA by sex, gross motor competency, PA parenting practices, and socioeconomic status.

**Results:**

Among 137 participants (54.0% female), average age was 13.6 months (SD = 1.7). MVPA was 72 ± 25 and 79 ± 26 minutes/day for females and males, respectively (*p* = 0.14). Total PA was 221 ± 48 and 238 ± 47 minutes/day for females and males, respectively (*p* = 0.04). Both gross motor competency and PA-encouraging parenting practices were positively associated MVPA (*p* = 0.01 and *p* = 0.02, respectively) and total PA (*p* = 0.02 and *p* = 0.01, respectively); however, these relationships did not differ by sex (*p* = 0.11 and *p* = 0.89, respectively). After accounting for gross motor competency and PA parenting practices, total PA was 15 minutes/day lower among females than males (*p* = 0.04).

**Conclusions:**

This cross-sectional study of US children observed a sex gap in total PA at 10–16 months of age. Gross motor development and PA parenting practices did not differ by child sex nor explain the sex difference in PA. A longitudinal investigation should follow to further narrow down when sex differences in PA emerge and to determine the factors that lead to this difference.

## INTRODUCTION

Physical activity (PA) is a key health behavior for preventing obesity, type 2 diabetes, and cardiovascular disease.^1^ However, physical inactivity is prevalent across US populations.^1^ Given that physical inactivity tends to track over time,^2^ it is especially alarming that a substantial proportion of preschoolers aged 3 and 4 years are not sufficiently physically active.^3–6^ In particular, lower PA has been observed in females than males across countries and socioeconomic classes.^7,8^ A pooled analysis of 14 studies found that on average females performed 17% less total PA than males in youth samples aged 4–18 years from western countries.^9^ A review^6^ of 36 studies demonstrated a clear sex gap in PA levels among preschool-aged children across many countries. However, the timing of when this sex gap begins is yet unclear as PA development at infancy and toddler ages has only recently begun to be investigated.^10^ Among the few existing studies, while an infant study reported no sex differences in PA,^11^ some toddler studies,^12^ but not all,^13,14^ found significant sex differences in PA. Taken together, the sex gap in PA may begin to emerge in late infancy or early toddler ages.

Understanding the factors underlying a sex gap in PA among young children is important to guide intervention strategies. Previous studies have investigated several possible explanations for the sex differences in PA among school-aged and older children, including developmental, parental, and environmental factors.^15–17^ At 1 year of age, most children are expected to reach important milestones for PA, such as walking and throwing a ball. Gross motor skills are viewed as the building blocks and foundation of these PA milestones.^18^ In an early childhood context, gross motor development is the development of larger muscles that enable a child to hold his/her head up, sit, crawl, and eventually walk, run, and skip. Gross motor skill competence has been shown to be associated with PA in early childhood,^19–21^ including in 2-year-old children.^22^ In addition, parents play a crucial role in setting young children’s routines, limits, and physical environment.^23^ A sex difference in parental support for child PA has been observed among older children.^15,24^ However, it is unknown whether a sex difference in PA, if existing, could be explained by gross motor development and PA parenting practices around 1 year of age when a child begins to walk independently.

To address these research gaps, the primary aim of this study was to compare PA levels between females and males at approximate age 12 months in a sample of children residing in the US Midwest area. We hypothesized that females are less active than males at approximate age 12 months. The exploratory aim was to investigate whether gross motor competency and PA parenting practices explain a sex difference in PA.

## METHODS

### Study Sample

The study sample was Child and Mother Physical Activity Study (CAMPAS) participants. CAMPAS is a longitudinal study aiming to develop an accelerometer-based activity recognition algorithm for toddlers and compare PA development trajectories from age 1 to 3 years between females and males. The eligibility criteria for child participants included being age 10 to 15 months at baseline assessment, having no cerebral palsy or other medical conditions precluding physical movement, and residing in the Chicago metropolitan area. The eligibility criteria for mother participants included being age 18 years or older, speaking and understanding English or Spanish, and living with the participating child at least 50% of the time. Recruitment flyers were distributed to various community organizations and locations, such as pediatric clinics, commercial indoor child playrooms, park districts, childcare centers, and libraries. Recruitment emails were also sent to potentially eligible individuals who were identified from a local healthcare patient electronic database. Detailed recruitment methods and study protocol are available in our previous publication.^25^ Participants were recruited between August 2022 and March 2024. Data collection was conducted in-person in multiple public locations across the Chicago metropolitan area (e.g., child indoor playrooms) as well as remotely. During a study visit, mothers completed a REDCap-based survey.

### Outcomes

Outcome variables included MVPA (minutes/day) and total PA (light or higher-intensity PA; minutes/day).^12,26^ These variables were derived from ActiGraph wGT3X-BT accelerometer data. Mothers were given verbal instructions on their child’s accelerometer wear during an in-person or virtual study visit. Participants received an accelerometer package that contained an accelerometer with an adjustable waist belt, an instruction sheet, a wear log sheet, and a prepaid envelope during an in-person visit or via United States Postal Service mail. Mothers were asked to assist their child in an accelerometer wear for 7 days and 24 hours and complete the wear log. Upon 7-day wear completion, mothers returned the accelerometer package via an in-person pickup by research staff or USPS mail. Accelerometer data were downloaded and reintegrated in a 15-second epoch using the ActiLife software version 6.14. Because an accelerometer data reduction standard for toddlers has not been established, we adopted the following data reduction method that were used in previous toddler and preschooler studies. We extracted data collected between 6 AM and 10 PM.^27–29^ Non-wear time, defined as ≥ 20 consecutive zero counts,^12,26,30,31^ was excluded. A valid wear day was defined as a day with ≥ 480 wear minutes of accelerometer data between 6AM and 10PM.^11,13,30^ At least 4 valid wear days were required to ensure ≥ 80% reliability.^13^ Participants who had fewer than 4 valid wear days were asked to re-wear an accelerometer.^13,26,32^ Light intensity was defined as 25–417 counts per 15 seconds and Moderate or higher intensity was defined as >417 counts per 15 seconds.^26,33^ Daily accumulated minutes spent in LPA and MVPA were calculated. Total PA was calculated by summing daily minutes spent in LPA and MVPA.^12,26^

### Exposures

The primary exposure of interest was biologically assigned sex, as reported by mothers in the study survey. The exposures for the exploratory aim were gross motor competency and PA parenting practices. Gross motor competency was assessed using mother-reported Ages and Stages Questionnaire (ASQ) gross motor subscale responses.^34^ ASQ gross motor subscale contained 6 items with three response options (yes = 10; sometimes = 5; not yet = 0). The item scores were summed to calculate a gross motor subscale score, which was used to assign the child into three categories: above the cutoff (the child’s development appears to be on schedule), close to the cutoff (the child needs learning activities and monitoring), and below the cutoff (the child may need further assessments with a professional).^34^ The cutoff scores were used as defined in ASQ scoring guides. In the current report, we combined “close to the cutoff” and “below the cutoff” categories as lower gross motor competency. We considered “above the cutoff” as higher gross motor competency.

Parenting practices regarding PA were assessed using the Preschooler Physical Activity Parenting Practices (PPAPP) questionnaire.^35^ Examples of the 16 PA-encouraging question items included: “How often do you… a) play active games with your child (such as playing ball or racing)?; b) take your child to the park?; c) go on a walk with your child?; and d) say positive things to motivate your child to be more active?” Examples of the 11 PA-discouraging question items included: “How often do you… a) allow your child to watch TV for long periods of time?; b) drive your child, when it was easy to walk?; c) tell your child he/she will get hurt if he/she plays actively?” Each item was rated in a 5-point Likert scale (1 = never; 2 = rare; 3 = sometimes; 4 = often; 5 = always). Scores from the 16 PA-encouraging parenting practices items were averaged. The PA-encouraging parenting practices score was then split at the median (lower PA encouraging: <3.7; higher PA encouraging: ≥3.7). The PA-discouraging parenting practices score was calculated by averaging the scores from three subscales: the 3-item Promote Screen Time subscale; the 3-item Promote Inactivity subscale; and the 5-item Psychological Control subscale. The PA-discouraging parenting practices score was then split at the median (lower PA-discouraging: <1.9; higher PA-discouraging: ≥1.9).

### Other Variables

To account for multi-dimensional Social Determinants of Health,^36^ we considered Child Opportunity Index (COI).^37^ COI is a composite index measured at a census tract level that captures neighborhood resources and conditions that matter for children’s healthy development.^37^ Using the COI 3.0 database matched with participants’ residential addresses, each participant was assigned to one of the five Chicago metropolitan COI categories: very low, low, moderate, high and very high. They were then recategorized into very low or low, moderate, and high or very high.

The following potential covariates were considered based on the literature: child’s racial and ethnic background, maternal education, maternal employment, maternal marital status, childcare attendance, and the presence of siblings in household.^11,12,30^ In the study survey, mothers reported child ethnicity and race (Hispanic, non-Hispanic Asian, non-Hispanic Black, non-Hispanic multi-race, vs. non-Hispanic White), childcare attendance (yes vs. no), maternal education (4-year college degree or higher vs. less than 4-year college), maternal employment (full-time, part-time vs. not employed), marital status (married vs. non-married), and the presence of young (0–5 years old) and old (6–17 years old) siblings (yes vs. no).

In addition, we considered two growth and developmental factors as potential covariates: weight-for-length percentile and ability to walk independently. Mothers provided a copy of the child’s most recent clinic visit summary that contained a date of visit as well as length and weight measurements. World Health Organization weight-for-length percentile^38^ was calculated based on the anthropometry measurements, which was then dichotomized into < 85 and ≥ 85th percentile.^39^ Mothers also reported whether the participating child was able to walk independently in the survey (yes vs. no).

### Power Consideration

Statistical power for the primary aim was calculated to detect an effect size of 0.5 standard deviation (SD) (e.g., 10 minutes/day of MVPA difference between males and females at a SD of 20 minutes/day). Priori power analysis indicated that a sample size of 126, with a goal of recruiting 50% female, would provide 80% power to detect a 0.5 SD effect size at a significance level of 0.05 using in two-sample t-test (two-sided).

### Statistical analysis

All analyses were conducted in SAS 9.4 (Cary, NC). A significance level was set at 0.05 (two-sided). Descriptive analyses were conducted. Normality of the outcome variables were evaluated using the Shapiro-Wilk tests. Bivariate analyses (i.e., t-tests and Analysis of Variance) were conducted to compare the PA outcome variables by sex, COI, gross motor competency, PA parenting practices, and potential covariates. To achieve the exploratory aim, we conducted Chi-square tests to compare the distribution of the gross motor and PA parenting practice categories between males and females.

To achieve the primary aim, multivariable linear regression analyses were conducted to compare MVPA and total PA by sex, gross motor competency, and PA parenting practice variables, adjusted for COI categories. We additionally included other potential covariates that were found to have p-value < 0.10 in bivariate analyses. We tested the interaction terms between sex and gross motor competency and between sex and PA parenting practices.

## RESULTS

A total of 137 participants (74 females; 54.0%) completed baseline assessment. Due to visit scheduling issues (e.g., rescheduling), 7 participants turned 16 months at baseline assessment. Average age of child participants was 13.6 months (SD = 1.7). Mothers’ average age was 34.5 years (SD = 4.2). None of the study variables had a missing value. Average number of valid monitor wear days was 6.6 days (SD = 0.9), and average monitor wear hours were 14.6 hours/day (SD = 1.6) between 6AM and 10PM. Average MVPA was 75 minutes/day (SD = 25), and average total PA was 229 minutes/day (SD = 48).

Table 1 presents PA levels by sex and other variables. On average, females engaged in 72 ± 25 minutes/day of MVPA compared to 79 ± 26 minutes/day for males (*p* = 0.14). Females on average engaged in 221 ± 48 minutes/day of total PA compared to males at 238 ± 47 minutes/day (*p* = 0.04). Twenty-two percent of the sample were categorized as lower gross motor competency. The following PA-supportive parenting practice items had an average score of 4 or higher (often or always): “go on a walk with the child”; “set aside time for active play”; “teach the child that being active is good for his/her health”; and “have outdoor toys available for the child” (data not shown).

### Results of the exploratory aim.

Children with higher gross motor competency had higher total PA, compared to those with lower gross motor competency (*p* = 0.003; Table 1). Children whose parents had higher PA-encouraging parenting practice scores had higher MVPA and total PA (*p* = 0.03 and *p* = 0.02, respectively), compared to those with lower scores. PA-discouraging parenting practice scores were not associated with MVPA or total PA. Sixteen percents of males and 27% of females were categorized as having lower gross motor competency; the proportions were not statistically significantly different (*p* = 0.11; Table 2). PA-encouraging and PA-discouraging parenting practices did not differ by sex (*p* = 0.93 and *p* = 0.75, respectively).

### Results of the primary aim.

After adjusting for the covariates, a sex difference in total PA was estimated as 15 minutes (*p* = 0.03; Table 3). A sex difference in MVPA was estimated as 6 minutes; however, it was not statistically significant (*p* = 0.16). Higher gross motor competency was associated with 13 minutes/day higher MVPA (*p* = 0.02) and 25 minutes higher total PA (*p* = 0.01). Higher PA-encouraging parenting practice scores were significantly associated with 11 minutes higher MVPA (*p* = 0.01) and 18 minutes higher total PA (*p* = 0.02). No significant interactions were found between sex and gross motor competency or between sex and PA parenting practices.

## DISCUSSION

### Main findings.

This cross-sectional study found that females on average engaged in 15 minutes/day less total PA than males in a US sample of children aged 10–16 months. MVPA also tended to be lower among females by 6 minutes/day; however, it did not reach a statistical significance. In this study sample, gross motor competency and PA parenting practices did not differ between males and females, which implies that gross motor competency and PA parenting practices do not explain the sex difference in PA. Regardless of sex, higher gross motor competency and higher PA-encouraging parenting practices were associated with higher PA.

### Physical activity among toddlers.

In the CAMPAS sample with relatively high maternal education levels (88% college graduates), MVPA was estimated 75 minutes/day and total PA was 229 minutes/day. The sample overall had higher MVPA levels, but similar total PA levels, compared to 60 minutes/day of MVPA (95% CI = 49, 72) and 246 minutes/day of total PA (95% CI = 190, 302) estimated in a meta-analysis of 16 toddler PA studies.^40^ This is one of the few studies to evaluate sensor-measured PA level at the age when children begin to walk independently. The finding of significantly lower PA in children from neighborhoods with very low or low COIs suggests that children residing in neighborhoods with less positive neighborhood resources may have limited PA opportunities to develop healthy PA behaviors from a very young age. This finding is aligned with a review^41^ that emphasized the importance of environmental factors such as outdoor environment and play spaces for PA promotion among young children. As the toddler PA research field continues to expand, future research is guaranteed to identify specific neighborhood resources that can promote PA from a young age.

### Sex differences in physical activity among toddlers.

Literature is well established to support that females are less active than males from preschool ages to adulthood. However, mixed findings have been reported in identifying the timing of when this sex difference emerges.^42^ Shull et al.^11^ reported no sex difference in PA in a US sample of 143 children aged 6–7 months. Hnatiuk et al.^13^ found no sex difference in MVPA and LPA in an Australian sample of 295 children at age 19 months. In contrast, Carson and Kuzik^12^ reported a 9 minutes/day lower MVPA among females in a Canadian sample of 149 children at age 19 months. In a US sample of 277 children aged 12–36 months, females were less active than males.^14^ The present study suggests that the sex difference in MVPA and total PA emerges at 10–16 months of age. Longitudinal studies are required to identify the exact timing of the sex difference in PA behavior development trajectory during early childhood. Future studies must also consider various important confounding factors, including individual, familiar, and neighborhood variables, to determine and explain sex differences in PA among young children.

### Gross motor development and physical activity among toddlers.

A few prior studies^21,43^ reported that gross motor skill competency was higher among females than males at preschool age. However, in the current toddler sample, gross motor competency did not significantly differ by sex, and it little explained the sex difference in total PA found in the sample. Regardless, the finding of a positive association between gross motor development and PA levels is consistent with the findings of prior studies^19–22^ and supports that gross motor development and PA are strongly linked in reciprocal feedback loops.^44^ This result suggests that gross motor development should be closely monitored and appropriate actions should be taken in the general toddler population to support establishing healthy PA development from an early child age.

### Physical activity parenting practices and physical activity among toddlers.

This study revealed no difference in PA parenting practices by child sex, consistent with prior study findings^45^ in UK children aged 2 years. This suggests that unlike for older children,^15,24^ a child’s sex may not play a role in determining parents’ PA practices for their children during toddlerhood. Instead, a sex difference in PA parenting practices may appear in later childhood.

PA parenting practices, such as role modeling and logistic support, have been shown to strongly influence a child’s PA behaviors.^46^ Consistent with the literature, this study found that PA-encouraging parenting practices, but not PA-discouraging parenting practices, were associated with toddlers’ MVPA levels. The most frequent PA-encouraging parenting practices included “go on a walk with the child”; “set aside time for active play”; “teach the child that being active is good for his/her health”; and “have outdoor toys available for the child”. These findings will inform PA promotion strategies for young children. For families with lower neighborhood and home resources (e.g., lack of park access or outdoor toys) and with less time for active play or walk, a childcare setting could provide great opportunities to offer PA-encouraging environments.^47^ Childcare PA policy has shown to have positive impacts on PA promotion among young children.^48^ Home- and childcare-based PA-encouraging practices are recommended to promote young children’s engagement in PA.

### Strengths and Limitations.

The strengths of the current study include sensor-based PA assessment and no missing information. The limitations include that the cross-sectional study design prevents us from detecting causality. Specifically, this study was investigated assuming that gross motor competency leads to PA development;^44^ however, PA could also have improved gross motor competency in feedback loops.^44^ Due to relatively high maternal education levels (88% college graduates), the study findings may not be generalizable to other populations.

## Conclusions

This cross-sectional study quantified 15 minutes/day lower total PA among females than males in a community-based convenience sample of US children aged 10–16 months. Gross motor development and PA-encouraging parenting practices were positively associated with PA among children aged 10–16 months. However, gross motor development or PA parenting practices did not explain the sex difference in PA. While these findings suggest that the sex difference in PA emerges during early toddlerhood, future longitudinal studies should be conducted to determine the exact timing of, as well as the factors that might drive, the sex difference in PA in young children.

## Figures and Tables

**Table 1:** Comparison of physical activity by sex and other participant characteristics

	Sample size	MVPA (minutes/day)	*p*	Total PA (minutes/day)	*p*
	n	mean ± SD		mean ± SD	
Total	137	75 ± 25	NA	229 ± 48	NA
Sex			0.14		0.04
Female	74	72 ± 25		221 ± 48	
Male	63	79 ± 26		238 ± 47	
Maternal education			0.83		0.74
< 4-year college degree	17	74 ± 33		225 ± 65	
≥ 4-year college degree	120	75 ± 24		229 ± 45	
Maternal employment			0.06		0.09
Full-time employed	79	71 ± 26		221 ± 49	
Part-time employed	23	82 ± 21		240 ± 34	
Not employed	35	81 ± 25		239 ± 50	
Maternal marital status			0.71		0.82
Married	113	75 ± 26		229 ± 48	
Not married		73 ± 22		227 ± 48	
Race and ethnicity			0.61		0.86
Hispanic	23	72 ± 30		226 ± 60	
Non-Hispanic Asian	12	81 ± 23		240 ± 49	
Non-Hispanic Black	11	78 ± 21		234 ± 47	
Non-Hispanic White	87	74 ± 25		226 ± 44	
Non-Hispanic other	4	89 ± 34		250 ± 59	
Presence of a sibling aged 0–5 years			0.03		0.02
Yes	68	80 ± 26		238 ± 47	
No	69	70 ± 24		219 ± 47	
Presence of a sibling aged 6–17 years			0.80		0.52
Yes	28	76 ± 29		234 ± 51	
No	109	75 ± 25		227 ± 47	
Childcare attendance			0.85		0.87
Yes	51	75 ± 25		228 ± 45	
No	86	75 ± 26		229 ± 50	
Media screen exposure			0.59		0.60
Yes	82	76 ± 27		231 ± 51	
No	54	74 ± 23		226 ± 43	
Child Opportunity Index			0.12		0.20
Very low or low	30	67 ± 27		215 ± 58	
Moderate	26	75 ± 28		230 ± 56	
Hight or very high	80	78 ± 23		234 ± 40	
Weight-for-height percentile			0.50		0.39
< 85th percentile	90	77 ± 26		233 ± 46	
≥ 85th percentile	38	74 ± 25		225 ± 47	
Walking independently			0.52		0.03
Yes	83	74 ± 23		236 ± 44	
No	54	77 ± 29		218 ± 51	
Gross motor competency			0.14		0.003
Higher	106	69 ± 27		206 ± 49	
Lower	30	77 ± 25		236 ± 46	
PA-encouraging parenting practices score			0.01		0.02
Higher	60	82 ± 25		239 ± 47	
Lower	77	70 ± 25		220 ± 47	
PA-discouraging parenting practices score			0.59		0.55
Higher	66	74 ± 25		226 ± 48	
Lower	71	76 ± 26		231 ± 48	

M ± SD, mean ± standard deviation; MVPA, moderate- and vigorous-intensity physical activity; PA, physical activity; NA, not applicable.

**Table 2:** Gross motor competency and physical activity parenting practices by sex

	Female	Male	*p*
	n (%)	n (%)	
Gross motor competency			0.11
Higher	53 (73)	53 (84)	
Lower	20 (27)	10 (16)	
PA-encouraging parenting practices score			0.89
Higher	32 (43)	28 (44)	
Lower	42 (57)	35 (56)	
PA-discouraging parenting practices score			0.82
Higher	31 (49)	35 (47)	
Lower	32 (51)	39 (53)	

PA, physical activity.

**Table 3:** Multivariable linear regression models for moderate- and vigorous-intensity physical activity and total physical activity

	MVPA (minutes/day)		Total PA (minutes/day)	
	Coefficient (95% CI)	*p*	Coefficient (95% CI)	*p*
Intercept	64 (52, 77)	< 0.01	192 (170, 214)	< 0.001
Sex				
Female	−6 (−14, 2)	0.16	−15 (−29, −1)	0.04
Male	Reference	NA	Reference	NA
Maternal employment				
Not employed	9 (−0.5, 18)	0.06	17 (0, 34)	0.05
Part-time employed	6 (−6, 17)	0.33	13 (−7, 33)	0.21
Full-time employed	Reference	NA	Reference	NA
Child Opportunity Index				
Very low or low	−12 (−22, −2)	0.02	−20 (−38, −1)	0.04
Moderate	−3 (−13, 8)	0.59	1 (−18, 20)	0.95
High or very high	Reference	NA	Reference	NA
Presence of a sibling aged 0–5 years				
Yes	6 (−2, 14)	0.15	16 (1, 31)	0.03
No	Reference	NA	Reference	NA
Walking independently				
Yes	−6 (−15, 3.1)	0.20	11 (−5, 28)	0.18
No	Reference	NA	Reference	NA
Gross motor competency				
Higher	13 (2, 23)	0.02	25 (6, 45)	0.01
Lower	Reference	NA	Reference	NA
PA-encouraging parenting practices score				
Higher	11 (3, 19)	0.01	18 (3, 32)	0.02
Lower	Reference	NA	Reference	NA

CI, confidence interval; MVPA, moderate- and vigorous-intensity physical activity; NA, not applicable; PA, physical activity.

## Data Availability

Data may be available upon request.

## List Of Abbreviations

CAMPAS: Child and Mother Physical Activity Study
COI: child opportunity index
LPA: light-intensity physical activity
MVPA: moderate- and vigorous-intensity physical activity
PA: physical activity
